# On the History of Syphilis in Europe

**Published:** 1911-09-23

**Authors:** Daniel Sennert

**Affiliations:** Professor of Medicine in the University of Witeberg, and Physician to the Elector of Saxony, 1656.


### Medical Antiquities.

### ON THE HISTORY OF SYPHILIS IN EUROPE.

(From the works of DANIEL SENNERT, M.D., Professor of Medicine in the University of Witeberg, and Physician to the Elector of Saxony, 1656.)

Of all the contagious diseases, after plague and leprosy, venereal disease easily holds the first place. This disease is called by various names, of which the most common is the "French disease," a name given because it was amongst the French that it first became known in Europe. For when Charles VIII. of France was warring with King Alphonso against Naples A.D. 1493-94, this disease first began to rage in the French camp and to become known, and on that account was first called the "French disease" by the Italians. For Antonio Benevenius doubtless refers to this in his book "on the causes of obscure diseases," A.D. 1496, where he remarks: "The disease is now no longer confined to Italy, but has spread over all Europe."

Other authors, without exception, agree that it was in the French camp and at this time that the disease began to be known. The French, however, to transfer the disgrace from themselves to the Italians, call it the "Italian and the Neapolitan disease," because it first began to be known in Naples; others, alleging that the disease was introduced to the French camp by the Spaniards, call it the "Spanish disease." Others again call it the "Indian disease." For writers on the Indies state that in the part of America which is called Florida, the disease has been epidemic for centuries past. Frascatorius even calls it syphilis, others great pox, because pustules and tubercles break out indiscriminately as in the other poxes.

Jerome Reusner, Franciscus Vallesius, and other more recent writers believed that the disease was known to the ancients, and thought that Hippocrates (in the words of Reusner), "not merely drew a sketch but painted a vivid picture when he enumerated various symptoms which are observed in venereal disease, as abscesses, suppurations, etc." As a matter of fact, Hippocrates, in this passage, never dreamt of venereal disease, but, in the opinion of Galen and all the critics, described the plague, as is confirmed by the clinical progress of the disease. For the disease described began with fever, unlike venereal disease, and was epidemic, whereas venereal disease can be conveyed by contagion alone. Paracelsus states that the disease first appeared in the years 1478-1480, but this account differs from all others. For these agree that the disease was not known in Europe before the year 1493, whereas it had been endemic in the West Indies for a long time before that date, and was brought thence by the Spaniards into Italy.

Now Christopher Columbus began the first crossing to the West Indies in September 1492, and spent nearly two years on his travels, returning to Spain in 1494. His soldiers were infected with the disease in the West Indies, and on their return served as mercenaries in the Italian expedition, and so spread the infection. Venereal disease is more common in some countries than in others. The reason of this it were odious to inquire. However, I am certain that it is less common in Germany than in any other country. Gabriel Fallopius states that James Carpus, the first to employ the inunction of mercury, became rich by this alone, and left a sum of 40,000 scudi in gold, besides silver. Capiuaccia states that he himself made 18,000 crowns from the treatment of venereal disease. For myself, I candidly confess that, during the thirty-four years that I have practised medicine in Witeberg, not, by divine blessing, without profit, I have not earned as many crowns as Capiuaccia did thousands, for treating this disease, so rare is it here.

